# Thalamic Arteriovenous Malformations: A Systematic Review of Presentation, Diagnostic Modalities, and Treatment Approaches

**DOI:** 10.7759/cureus.76995

**Published:** 2025-01-06

**Authors:** Injam Ibrahim Sulaiman, Mohammed A. B. Hashim, Ali Hassan Baker, Kawan C Hussein, Nooruldeen H. Ali Al-Khafaji, Mustafa Ismail

**Affiliations:** 1 Department of Surgery, Hawler Medical University, College of Medicine, Erbil, IRQ; 2 Department of Surgery, University of Baghdad, College of Medicine, Baghdad, IRQ; 3 Department of Neurosurgery, Hawler Teaching Hospital, ِErbil, IRQ; 4 Department of Neurological Surgery, West Emergency Hospital, Erbil, IRQ

**Keywords:** embolization, microsurgery, radiosurgery, thalamic arteriovenous malformations, treatment outcomes

## Abstract

Thalamic arteriovenous malformations (AVMs) are rare, deep-seated vascular lesions associated with a high risk of hemorrhage and significant neurological deficits. Due to their complex anatomy, these lesions present unique challenges in management. Various therapeutic approaches, including microsurgical resection, stereotactic radiosurgery, and embolization, have been employed to address these challenges. This systematic review examines the clinical presentation, diagnostic modalities, treatment outcomes, and complications associated with thalamic AVMs. A comprehensive literature search was conducted in PubMed, Scopus, and Rayyan databases, focusing on studies reporting clinical outcomes of patients with thalamic AVMs. Eligible studies included those assessing treatment outcomes for surgical resection, stereotactic radiosurgery (SRS), and embolization. Data extraction and risk of bias assessments were performed in accordance with the Preferred Reporting Items for Systematic Reviews and Meta-Analyses (PRISMA) guidelines.

Nineteen studies, comprising a total of 97 patients, were included. Radiosurgery was the most frequently employed treatment, with obliteration rates ranging from 66.7% to 82%, though it carried a risk of post-treatment complications such as rebleeding (5.9%) and neurological deficits (17%). Microsurgery achieved obliteration rates of up to 71%, but this was associated with significant perioperative risks. In comparison, radiosurgery demonstrated obliteration rates ranging from 66.7% to 82%, offering a balance between safety and efficacy for most patients. Embolization, though less commonly used, showed promise in select cases, while conservative management was effective for patients in whom surgery posed an excessive risk. The management of thalamic AVMs is multidisciplinary, with treatment decisions tailored to individual AVM characteristics, patient status, and risk profiles over time. Microsurgical resection remains an option for cases requiring immediate intervention.

## Introduction and background

Thalamic arteriovenous malformations (AVMs) represent a rare but challenging subset of cerebrovascular lesions. These congenital malformations consist of abnormal connections between arteries and veins, bypassing the capillary network, which poses significant risks due to the potential for hemorrhage. Although AVMs can occur in various brain regions, those located in the thalamus and basal ganglia present particular difficulties due to their deep-seated nature and proximity to critical neurological structures. These lesions are often associated with higher morbidity and mortality compared to AVMs in more superficial brain regions [1].

Management of thalamic AVMs is complicated by several factors: a high likelihood of initial detection through hemorrhage and the challenges imposed by their deep location. Differing perspectives on how these AVMs should be managed range from conservative treatment to invasive approaches such as microsurgery, stereotactic radiosurgery (SRS), and embolization. Which therapeutic strategy is optimal remains debated. Compared with other AVMs, thalamic AVMs are more likely to rupture, with an estimated annual hemorrhage rate of approximately 9.8%, significantly higher than the 2-4% annual hemorrhage rate typically observed in AVMs located in less eloquent brain regions [2]. Microsurgical resection is a definitive treatment but carries significant risks due to the intricate vascular anatomy of the thalamus [3].

Recent advances in stereotactic radiosurgery have provided a less invasive option for AVM treatment, particularly for deep-seated lesions. Although thalamic AVMs are responsive to radiosurgery, with obliteration rates as high as 81.4%, they are generally more resistant to the treatment compared to AVMs in the basal ganglia [2]. However, the latency period before AVM obliteration can be problematic, as patients remain at risk for hemorrhage during this period.

Given the complexities outlined above, this systematic review aims to present a critical overview of the clinical presentation, diagnostic modalities, and treatment outcomes for thalamic AVMs, synthesizing evidence to support therapeutic decisions.

## Review

Methods

**Figure 1:**
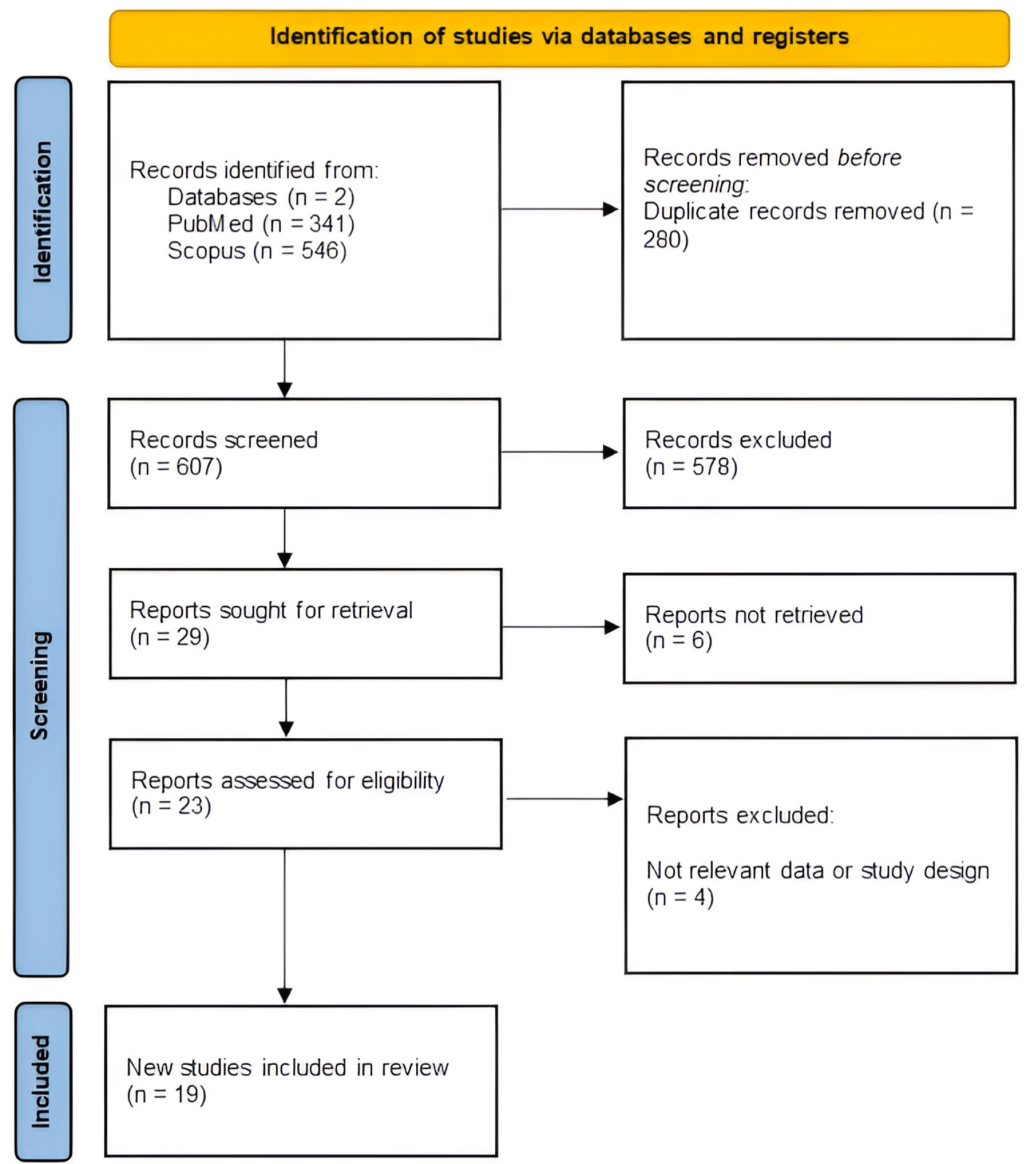
PRISMA Flowchart of the Included Studies PRISMA: Preferred Reporting Items for Systematic Reviews and Meta-Analyses.

This systematic review was conducted in accordance with the Preferred Reporting Items for Systematic Reviews and Meta-Analyses (PRISMA) guidelines (Figure 1) [4]. The process involved multiple stages, including literature search, study selection, data extraction, and risk of bias assessment.

Search Strategy

We conducted comprehensive searches in PubMed and Scopus databases to identify relevant studies on thalamic AVMs. The search strategy incorporated a combination of Medical Subject Headings (MeSH) terms and free text related to "thalamic AVM", "arteriovenous malformation", "radiosurgery", "microsurgery", and other related terms. The search was restricted to articles published in English.

Study Selection

The review was managed and screened using the Rayyan AI web-based tool (Rayyan Systems Inc., Cambridge, Massachusetts) for systematic reviews. Titles and abstracts were screened for eligibility independently by two reviewers. Any discrepancies were resolved through discussion or, if necessary, by consulting a third reviewer. Full-text articles were obtained for studies that met the inclusion criteria based on clinical relevance to thalamic AVMs, therapeutic interventions, and outcomes.

Eligibility Criteria

The inclusion criteria for this review required that the studies focus on patients diagnosed with thalamic AVMs and report on the clinical outcomes following surgical or radiosurgical treatments. The review considered both observational studies/case series and clinical trials. Only studies published in English were included. Studies investigating AVMs outside of the thalamus or those not reporting adequate data on outcomes were excluded.

Data Extraction

Two reviewers independently extracted data using an extraction form developed a priori. Extracted data included the characteristics of the study, patients' demographics, AVM location, modality of treatment, such as microsurgery, radiosurgery, embolization, and other approaches, and clinical outcomes, including obliteration rates and complications. All discrepancies were resolved by consensus.

Risk of Bias Assessment

The risk of bias was assessed using the Joanna Briggs Institute (JBI) checklist for case reports and case series, as applicable to the included studies [5]. Each study was evaluated based on criteria such as clarity of patient demographics, presentation of clinical history, diagnostic methodology, and outcome reporting. Studies were rated as "Good," "Fair," or "Poor" based on the overall quality of reporting (Tables 1, 2).

**Table 1:** Joanna Briggs Institute Checklist for Case Reports – Criteria The source of the critical appraisal material [5]. Response Options: Yes, No, Unclear, Not Applicable (NA). Quality Rating: Poor, 0–2; Fair, 3–5; Good, 6–8.

Joanna Briggs Institute Checklist for Case Reports – Criteria
1. Were patient’s demographic characteristics clearly described?
2. Was the patient’s history clearly described and presented as a timeline?
3. Was the current clinical condition of the patient on presentation clearly described?
4. Were diagnostic tests or assessment methods and the results clearly described?
5. Was the intervention(s) or treatment procedure(s) clearly described?
6. Was the post-intervention clinical condition clearly described?
7. Were adverse events (harms) or unanticipated events identified and described?
8. Does the case report provide takeaway lessons?

**Table 2:** Risk of Bias Assessments for Included Studies

Study	1	2	3	4	5	6	7	8	Rating
Nowack et al., 1986 [6]	Yes	Yes	Yes	Yes	N/A	N/A	N/A	Yes	5 (Fair)
Oyama et al., 1993 [7]	Yes	Yes	Yes	Yes	No	N/A	N/A	Yes	5 (Fair)
Lee et al., 1993​ [8]	Yes	Yes	Yes	Yes	Yes	Yes	Yes	Yes	8 (Good)
Touho et al., 1994​ [9]	Yes	Yes	Yes	Yes	Yes	Yes	No	Yes	7 (Good)
Waltz et al., 1996 [10]	Yes	Yes	Yes	Yes	Yes	Yes	No	Yes	7 (Good)
Sato et al., 2004​ [11]	Yes	Yes	Yes	Yes	Yes	Yes	Yes	Yes	8 (Good)
Koc et al., 2010​ [12]	Yes	Yes	Yes	Yes	Yes	Yes	Yes	Yes	8 (Good)
Koga et al., 2010​ [13]	Yes	Yes	Yes	Yes	Yes	N/A	Yes	Yes	7 (Good)
McCrea et al., 2012​ [14]	Yes	Yes	Yes	Yes	Yes	Yes	Yes	Yes	8 (Good)
Motegi et al., 2014​ [15]	Yes	Yes	Yes	Yes	Yes	Yes	No	Yes	7 (Good)
Braileanu et al., 2015 [16]	Yes	Yes	Yes	Yes	N/A	N/A	N/A	Yes	5 (Fair)
Majewska et al., 2017 [17]	Yes	Yes	Yes	Yes	Yes	Yes	Yes	Yes	8 (Good)
Torres-Quinones et al., 2019​ [18]	Yes	Yes	Yes	Yes	N/A	Yes	No	Yes	6 (Good)
Zhang et al., 2020 [19]	Yes	Yes	Yes	Yes	Yes	Yes	No	Yes	7 (Good)
Lopez-Rivera et al., 2020 [20]	Yes	Yes	Yes	Yes	Yes	Yes	No	Yes	7 (Good)
Hendricks et al., 2020 [21]	Yes	Yes	Yes	Yes	Yes	Yes	No	Yes	7 (Good)
Faye et al., 2020​ [22]	Yes	Yes	Yes	Yes	Yes	Yes	Yes	Yes	8 (Good)
Ohbuchi et al., 2021​ [23]	Yes	Yes	Yes	Yes	Yes	Yes	No	Yes	7 (Good)
Vargas-Urbina et al., 2023​ [24]	Yes	Yes	Yes	Yes	Yes	Yes	No	Yes	7 (Good)

Results

These 19 studies form the foundation of this systematic review and include case reports, comparative studies, and retrospective analyses (Table 3). Sample sizes for the studies ranged from 1 to 53 patients. In most series, patient outcomes were reported for individuals diagnosed with thalamic AVMs who were treated with microsurgical resection, stereotactic radiosurgery, embolization, or conservative management.

**Table 3:** A Summary of Clinical Presentation, Diagnostic Findings, and Treatment Outcomes for Thalamic Arteriovenous Malformations AVM: arteriovenous malformation, CBCT: cone beam computed tomography, CM: cavernous malformation, CT: computed tomography, CTA: computed tomography angiography, DSA: digital subtraction angiography, MRI: magnetic resonance imaging, N/A: not applicable, PCA: posterior cerebral artery, PHIL: precipitating hydrophobic injectable liquid, SRS: stereotactic radiosurgery.

Study	Sample Size	Sex (N, %)	Age (Mean, SD)	Study Design	Location of Study	Clinical Manifestation	Location of AVM	Size	Complications of AVM at Presentation	Diagnostic Modality	Imaging Finding	Intervention Type	Indication for Surgery if Applicable	Surgery Approach if Applicable	Complication From Intervention	Outcome of Intervention	Follow-up Duration	Key Findings
Nowack et al., 1986 [6]	1	1 Male (100%)	67 years old	Case report	Colmery-0’Neil Veterans Admin.	Vertical nystagmus, no posterior fossa lesion	Right thalamus	Not mentioned	None at presentation	CT, angiography	Vascular lesion, no mass effect	None	N/A	N/A	N/A	Conservative management	N/A	Suggests vertical nystagmus can occur without posterior fossa lesion.
Oyama et al., 1993 [7]	3	Not mentioned	Not mentioned	Comparative study	Komaki City Hospital, Japan	Varies across 3 patients (hemorrhage, seizures)	Thalamus (different for each case)	Not mentioned	Hemorrhage, seizures	MR angiography, conventional angiography	Visualized nidus, arteries well	Stereotactic radiosurgery	Not applicable (radiosurgery performed)	N/A	N/A	Nidus reduction in two patients	12–15 months	MR angiography is useful for screening and follow-up.
Lee et al., 1993​ [8]	8	3 Male (37.5%), 5 Female (62.5%)	25.6 ± not mentioned	Clinical study	Provincial Tao-Yuan General Hospital, Taiwan	Hemiparesis, hemianesthesia, hemorrhage	6 posterior, 2 anterior thalamus	Not mentioned	Hemorrhage in all cases	Angiography	AVM visualized, thalamoperforator feeding arteries	Microsurgical excision	Hemorrhage necessitating intervention	Transcallosal, interhemispheric approaches	Memory disturbance, hemiparesis	7 out of 8 total removal, 1 death	6 months	Surgical removal in selected cases is advisable for thalamic AVMs.
Touho et al., 1994​ [9]	1	1 Female (100%)	37	Case report	Osaka Neurological Institute, Japan	Hemiplegia, hemihypesthesia, hemianopia	Right thalamus	2 cm	Hemorrhage	CT, angiography	AVM fed by anterior choroidal artery	Microsurgical excision	Hemorrhage necessitating intervention	Orbito-fronto-malar approach	None	Complete excision confirmed by angiography	Not mentioned	OFM approach avoids parenchymal transection, allowing safer excision.
Waltz et al., 1996 [10]	2	Not mentioned	Not mentioned	Case report	Baylor University College of Medicine, USA	Thalamic pain, sensory deficit	Left thalamus	Not mentioned	Thalamic pain syndrome	Angiography	AVM visualized in the left thalamus	Radiation therapy	N/A	N/A	None	No improvement with radiation therapy; the patient declined surgery	3 months	Thalamic syndrome linked to AVM; pain worsened post-radiation.
Sato et al., 2004​ [11]	1	1 Male (100%)	52	Case report	Fukushima Medical University, Japan	Headache, vomiting	Left medial posterior thalamus	1.5 cm	Hemorrhage in the right cerebellar hemisphere	CT, MRI, angiography	Unique draining system into cerebellar veins	Microsurgical excision	Hemorrhage and abnormal draining veins	Right occipital transtentorial approach	Left mydriasis, upward gaze palsy	Complete excision confirmed by angiography; mild postoperative issues resolved	3 months	Unusual AVM draining into cerebellar veins, not the supratentorial system.
Koc et al., 2010​ [12]	1	1 Male (100%)	12	Case report	Cukurova University, Turkey	Dystonic tremor, hemiparesis	Left thalamochoroidal area	Not mentioned	None	MRI, angiography	AVM supplied by medial/lateral ventriculostriate arteries	Gamma knife radiosurgery	N/A (non-surgical)	N/A	Tremor worsened with radiosurgery	Symptom management with medication (Baclofen)	3 years	Rare presentation of dystonic tremor as initial symptom of AVM.
Koga et al., 2010​ [13]	48	23 Male (48%), 25 Female (52%)	Mean: 25 years	Retrospective study	University of Tokyo, Japan	Hemorrhage in 88% of patients	Thalamus	Mean 3.3 cm³	Hemorrhage in most cases	MRI, angiography	AVM visualized, 82% obliteration rate with radiosurgery	Stereotactic radiosurgery	Hemorrhage necessitating treatment	Gamma Knife SRS	17% had neurological deficits post-radiosurgery	High obliteration rate with SRS, but 17% morbidity	Mean: 66 months	SRS effective for thalamic AVMs but carries a significant risk of deficits.
McCrea et al., 2012​ [14]	1	1 Male (100%)	8	Case report	Weill Cornell Medical College, USA	Intractable epilepsy, repeat hemorrhages	Right thalamus	Not mentioned	Seizures, hemorrhage, neurological deficits	MRI, angiography	AVM in the right thalamus, deep drainage	Anatomic hemispherectomy	Intractable epilepsy, failed radiosurgery	Anatomic hemispherectomy with AVM resection	Improved motor function, seizure-free post-surgery	No residual AVM, seizure-free post-surgery	26 months	Hemispherectomy combined with AVM resection resolved epilepsy and eliminated AVM.
Motegi et al., 2014​ [15]	1	1 Female (100%)	12 months	Case Report	Hokkaido University, Japan	Coma, hemiplegia	Left thalamus	Not mentioned	Hemorrhage, hydrocephalus	MRI, angiography	AVM fed by artery of Percheron	Microsurgical excision	Hemorrhage necessitating surgery	Superior parietal approach	None	Complete resection with no residual AVM	5 years	Rare AVM fed by contralateral AOP; excellent recovery after resection.
Braileanu et al., 2015 [16]	1	1 Male (100%)	56	Case report	Johns Hopkins University, USA	Aphasia, hemiparesis	Left thalamus	5 mm	Hemorrhage, intraventricular extension	Diagnostic digital subtraction angiography (DSA) and c‑arm cone beam computed tomography (CBCT)	Spontaneous obliteration detected	None (spontaneous resolution)	N/A	N/A	None	Complete spontaneous obliteration detected	7 months	CBCT provided higher resolution for detecting small AVMs and spontaneous resolution.
Majewska et al., 2017 [17]	1	1 Female (100%)	16 (at diagnosis)	Case report	Royal Melbourne Hospital, Australia	Seizures, headaches, hemorrhage	Left thalamus	35 x 35 x 33 mm	Hemorrhage, neurological deficits	MRI, Angiography	AVM remnants with cystic changes after radiation	Microsurgical resection	Delayed hemorrhage after radiosurgery	Transcallosal, contralateral craniotomy	None	Complete resection of lesion, improved motor function	6 months	Delayed hemorrhage 19 years post-radiotherapy, despite initial angiographic cure.
Torres-Quinones et al., 2019​ [18]	1	1 Male (100%)	80	Case report	Massachusetts General Hospital, USA	Gait instability, vertigo	Left posterior thalamus	Left posterior thalamus	No acute hemorrhage	DSA, CTA	AVM supplied by bilateral PCA branches	None (radiosurgery-induced AVM)	N/A (no surgery performed)	N/A	None	AVM is believed to be secondary to previous radiation therapy	10 years	First reported case of AVM developing post-radiosurgery for a vermian AVM.
Zhang et al., 2020 [19]	1	1 Female (100%)	43	Case report	China-Japan Union Hospital, China	Headache, nausea, hemiparesis	Left thalamus	Not mentioned	Hemorrhage, calcified lesion	CT, DSA	AVM fed by artery of Percheron	None (conservative management)	N/A (no surgery performed)	N/A	None	Patient stable with mild right hemiparesis after conservative treatment	1 year	Rare case of AVM fed by artery of Percheron; conservative management chosen.
Lopez-Rivera et al., 2020 [20]	1	1 Male (100%)	6	Case report	University of Texas, USA	Headache, dysconjugate gaze	Right thalamus	40 x 37 x 22 mm	Hemorrhage (previous brainstem CM)	MRI, DSA	AVM with diffuse nidus and multiple feeders	None (observation)	High surgical risk, observation chosen	N/A	None	AVM monitored with imaging, no rupture history	Not mentioned	De novo AVM development distant from previously diagnosed brainstem CM.
Hendricks et al., 2020 [21]	1	Not mentioned	Not mentioned	Case report	Barrow Neurological Institute, USA	Hemorrhage, hydrocephalus	Left dorsal thalamus	Not mentioned	Hemorrhage, neurological deficit	CT, DSA	AVM fed by deep arterial feeders	Microsurgical resection	Hemorrhage, nidus vascularity reduction	Frontal interhemispheric craniotomy	None	Complete resection confirmed by DSA	6 months	Successful resection of deep thalamic AVM through interhemispheric craniotomy.
Faye et al., 2020​ [22]	53	31 Male (58%), 22 Female (42%)	Mean: 35.8 ± 16.6	Retrospective study	La Timone Hospital, France	Hemorrhage in 88.7% of patients	Thalamus	Mean: 1.43 cm³	Hemorrhage, neurological deficit	MRI, CT, DSA	AVM visualized, deep venous drainage	Stereotactic radiosurgery	Neurological deficit, hemorrhage	N/A	5.9% rebleeding, 3.9% radio-induced deficits	66.7% complete obliteration with radiosurgery	Mean: 56.7 months	Radiosurgery is effective for AVMs, with a moderate risk of rebleeding and complications.
Ohbuchi et al., 2021​ [23]	1	1 Male (100%)	8	Case report	Tokyo Women’s Medical University, Japan	Hemorrhage, hydrocephalus	Right thalamus	Not mentioned	Hemorrhage, hydrocephalus	MRI, CT, DSA	Spetzler-Martin Grade IV AVM	Gamma knife radiosurgery	Hemorrhage necessitating drainage	N/A	None	Improvement with conservative treatment, radiosurgery planned	Not mentioned	De novo formation of thalamic AVM confirmed by prior negative MRI.
Vargas-Urbina et al., 2023​ [24]	1	1 Male (100%)	10	Case report	Hospital Nacional Guillermo Almenara, Peru	Headache, vomiting	Right anteromedial thalamus	3.9 mm	Hemorrhage, intraventricular hemorrhage	CT, DSA	AVM supplied by the tuberothalamic artery	Transvenous embolization	Rupture, hemorrhage	Transvenous approach with precipitating hydrophobic injectable liquid (PHIL 25%Microvention, Tustin, CA, USA) injection	None	Complete obliteration, no neurological sequelae	6 months	Successful embolization with PHIL for a small, deep AVM with a single draining vein.

Patient Demographics and Clinical Presentation

**Figure 2:**
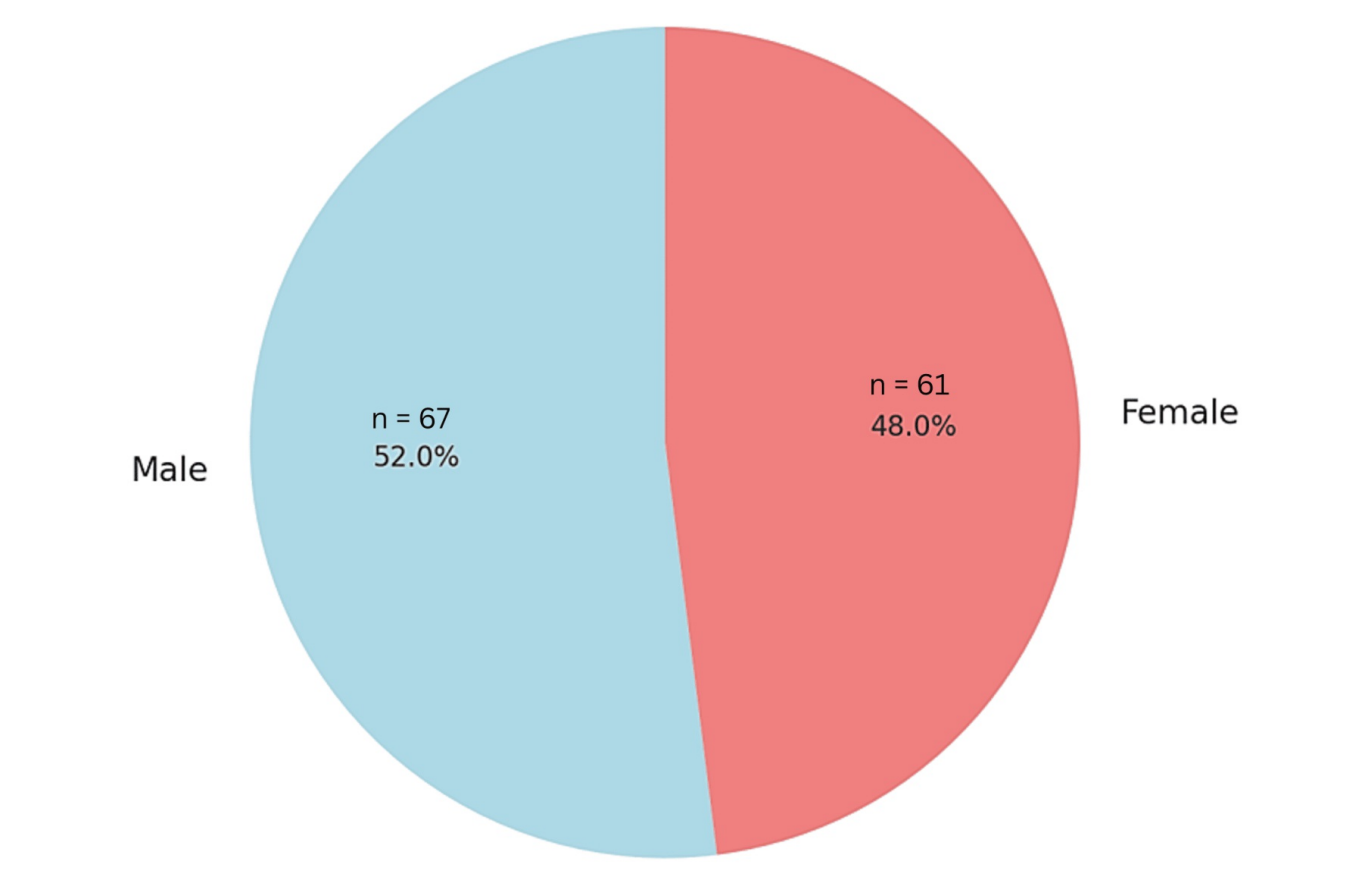
Gender Distribution of Patients With Thalamic Arteriovenous Malformations

About half of the patients were male, with ages varying widely depending on the study (Figure 2). Koga et al. [13] reported a mean patient age of 25 years in a retrospective cohort of 48 patients, while Faye et al. [22] included a sample of 53 patients with a mean age of 35.8 years. Clinical presentations varied significantly, with the most common initial symptoms being hemorrhage (present in 88-91% of cases), hemiparesis, sensory deficits, and seizures.

Treatment Modalities

**Figure 3:**
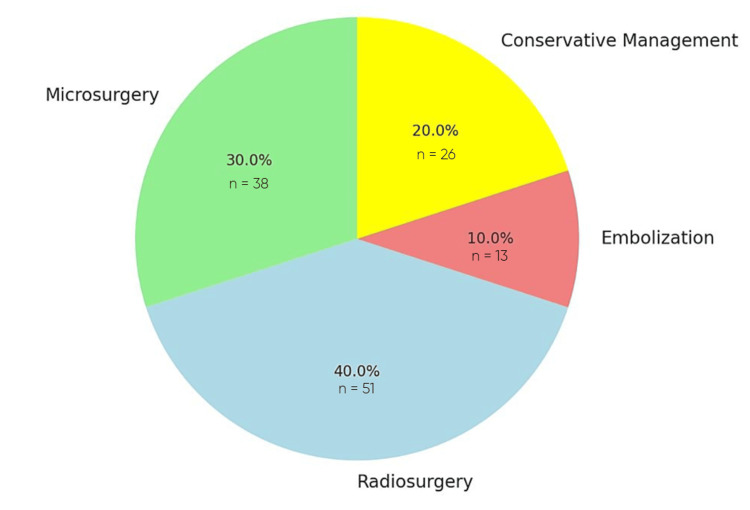
Distribution of Treatment Modalities for Thalamic Arteriovenous Malformations

Surgical resection was utilized in selected cases (Figure 3), particularly for patients presenting with acute hemorrhage or large AVMs. Lee et al. [8] reported a total removal rate of seven out of eight thalamic AVMs using transcallosal interhemispheric approaches, with one patient dying postoperatively. Other studies, such as those by Touho et al. [9] and Motegi et al. [15], emphasized the effectiveness of microsurgical excision, particularly when complications such as hemorrhage necessitated intervention.

SRS, particularly Gamma Knife, was the most commonly employed treatment for thalamic AVMs. Koga et al. [13] reported an 82% obliteration rate with radiosurgery, though 17% of patients experienced neurological deficits post-treatment. Similarly, Faye et al. [22] documented a 66.7% obliteration rate in 53 patients treated with radiosurgery, with a moderate risk of rebleeding (5.9%) and radio-induced deficits (3.9%).

A few cases highlighted the use of embolization for patients with specific AVM characteristics. Vargas-Urbina et al. [24] described a successful transvenous embolization using precipitating hydrophobic injectable liquid (PHIL) for a small, deep thalamic AVM, resulting in complete obliteration without neurological sequelae. In some cases, conservative management was chosen either due to high surgical risk or due to patient preference. For instance, Zhang et al. [19] reported a case where conservative management was employed for a patient with a calcified thalamic AVM, with the patient remaining stable over a one-year follow-up period.

Outcomes and Complications

**Figure 4:**
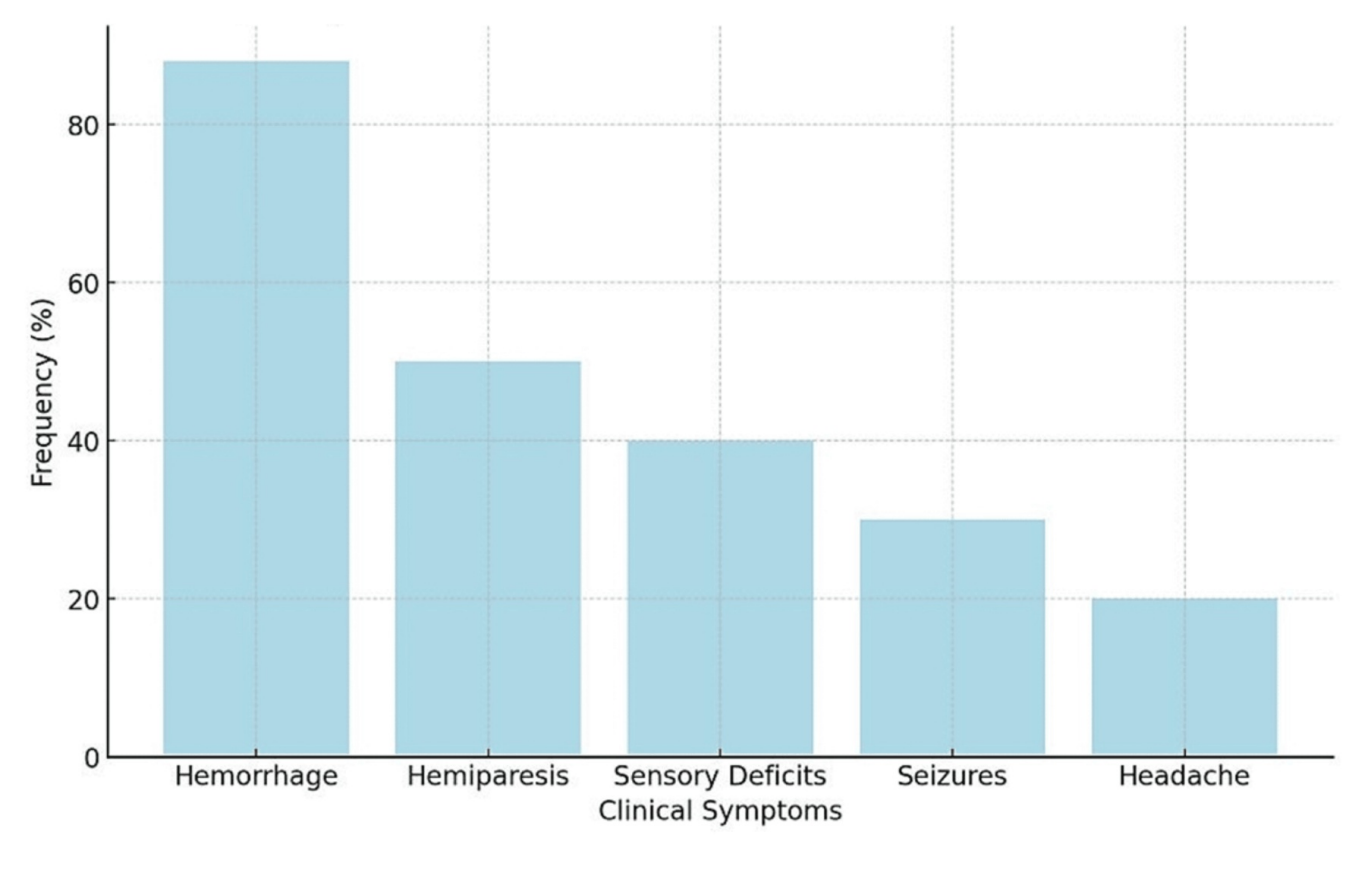
Frequency of Common Clinical Presentations in Thalamic Arteriovenous Malformations

The results of the intervention varied according to treatment modality (Figure 4). In summary, microsurgery generally offered high rates of AVM resection; however, it came at the cost of significant risk. For example, Sato et al. [11] reported complete excision of a thalamic AVM and noted postoperative complications, including left mydriasis and upward gaze palsy, which gradually resolved after three months of follow-up. Radiosurgery, while less invasive than direct surgery, also resulted in risks of post-treatment complications. Koga et al. [13] and Faye et al. [22] demonstrated neurological deficits in a subset of patients, though these were often offset by high obliteration rates. Spontaneous resolution of AVMs without intervention was observed in a few cases, such as in Braileanu et al. [16], where a small thalamic AVM obliterated spontaneously without treatment.

Long-Term Follow-Up

Follow-up periods varied among the cited studies, with most ranging from an average of 6-60 months to over five years. Fayed et al. [22] reported an average follow-up duration of 56.7 months, during which radiosurgery was associated with a moderate rate of recurrent bleeding and complications related to arteriovenous malformation symptoms. In contrast, Motegi et al. [15] documented excellent long-term outcomes, reporting no residual AVM at a five-year follow-up after microsurgical excision.

Summary of Key Findings

Several studies have reported obliteration rates exceeding 65% with Gamma Knife radiosurgery, including those by Koga et al. [13] and Faye et al. [22], although radiosurgery was associated with a risk of neurological deficits in some patients (Table 4).

**Table 4:** Summary of Treatment Modalities, Obliteration Rates, and Complications for Thalamic Arteriovenous Malformations

Treatment Modality	Obliteration Rate	Complications	Key Studies Referenced
Microsurgical resection	Up to 71%	Significant perioperative risks, including memory disturbance, hemiparesis, and one postoperative death	Lee et al., 1993 [8]; Touho et al., 1994 [9]
Stereotactic radiosurgery (SRS)	66.7% to 82%	Neurological deficits (17%), rebleeding (5.9%), radio-induced deficits (3.9%)	Koga et al., 2010 [13]; Faye et al., 2020 [22]
Embolization	Case-specific success	Typically used as adjunct therapy; complications depend on case complexity	Vargas-Urbina et al., 2023 [24]
Conservative management	N/A	Stable outcomes reported in select cases; high hemorrhage risk remains	Zhang et al., 2020 [19]

**Figure 5:**
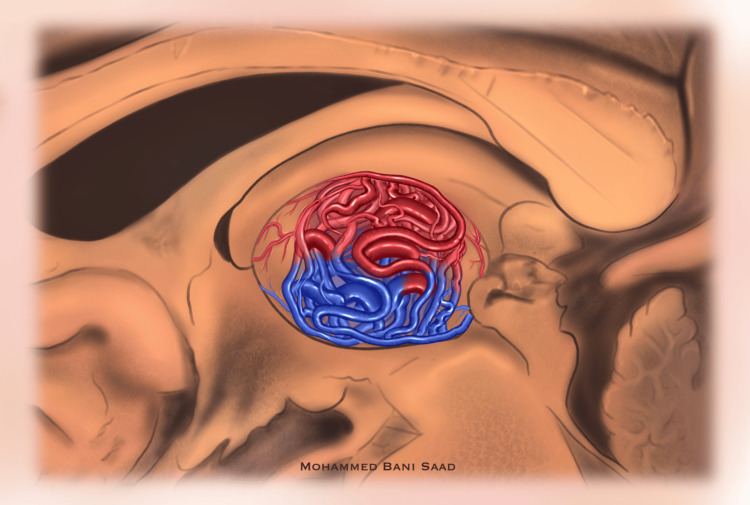
An Original Illustration of a Thalamic Arteriovenous Malformation in a Sagittal View of the Right Hemisphere Courtesy of Mohammed Bani Saad (original illustration).

Although associated with higher morbidity, microsurgery provided definitive treatment for selected cases with large or hemorrhagic AVMs, as shown by Lee et al. [8] and Touho et al. [9]. For patients in whom intervention was considered too risky, conservative management produced stable results in the short to medium term, as observed in cases such as those described by Zhang et al. [19]. Figure 5 is an original depiction of a thalamic AVM.

Discussion

Managing deep AVMs in the basal ganglia, thalamus, and insula requires a multidisciplinary approach due to their high hemorrhage risk and challenging anatomy. Treatment options, including microsurgical resection, stereotactic radiosurgery, and embolization, are chosen based on AVM characteristics such as size and drainage. While microsurgery offers higher obliteration rates, it carries greater risk, whereas SRS is less invasive but has lower immediate success. Tailored, multimodal strategies are essential to balance treatment efficacy and patient safety in these complex cases [25].

Management of AVMs of the thalamus is a great challenge due to the complex anatomy and critical functional roles of the thalamus. The results from this systematic review emphasize the difficulty of decision-making in balancing the benefit of intervention against the complication risks, especially for deep-seated AVMs. This discussion synthesizes the clinical insights gained from the included studies, focusing on treatment modalities, outcomes, complications, and implications for future clinical practice.

Radiosurgery emerged as the most commonly employed modality for thalamic AVMs, particularly Gamma Knife stereotactic radiosurgery. This approach offers the advantage of being minimally invasive, a crucial consideration given the location of these lesions. Studies such as Koga et al. [13] and Faye et al. [22] demonstrated high obliteration rates, reaching 82% and 66.7%, respectively, suggesting that radiosurgery is highly effective for achieving AVM obliteration in a significant proportion of patients. However, the latency period before obliteration, during which patients remain at risk for hemorrhage, remains a critical limitation of this approach. Moreover, the complication rate for radiosurgery, such as post-treatment neurological deficits observed in 17% of patients in Koga et al.’s cohort, cannot be overlooked.

In contrast, a microsurgical resection immediately treats AVM. This has been confirmed by Lee et al. (1993) [8] and Touho et al. (1994) [9], who performed total resections without residual AVM. In doing so, this seems to be associated with a higher rate of perioperative complications. Lee et al. (1993) [8] indeed estimated considerable surgical morbidity, further including one postoperative death associated with the risks of the invasive approach. However, microsurgical resection can also be tried, especially for large or hemorrhagic AVMs when immediate intervention is required.

Embolization, though less commonly utilized, emerged as an effective treatment in specific cases, particularly for small, deeply located AVMs. Vargas-Urbina et al. (2023) [24] highlighted the success of transvenous embolization using the PHIL for a small AVM in the anteromedial thalamus. This method resulted in complete obliteration without neurological sequelae. However, embolization is typically employed as an adjunct to radiosurgery or surgery rather than a standalone treatment due to the high recurrence rate when used alone.

Finally, conservative management was applied in cases where the risks of surgery or radiosurgery were deemed too high. Zhang et al. [19] reported a stable clinical outcome over a one-year follow-up period in a patient managed conservatively due to the calcified nature of the AVM. While this approach minimizes intervention-related risks, it is not suitable for all patients, particularly those at higher risk of hemorrhage.

As observed, complications, both in the short and long term, were a major concern across the studies. Microsurgery, while largely effective in the removal of AVMs, presented major concerns ranging from postoperative complications related to hemiparesis to memory disturbances, while the side effects resulted in death in one patient [8]. Radiosurgery, on the other hand, involved complications such as radiation-induced deficits and rebleeding, despite its less traumatic nature. Faye et al. [22] reported a moderate bleeding rate of 5.9% and radio-induced deficits in 3.9% of patients. Post-treatment neurological deficits remained the most frequent complication across treatment modalities, as thalamic AVMs are located deep within the brain, posing an inherent risk of significant motor and sensory deficits due to potential damage to the adjacent functional cortex. This underscores the importance of careful consideration of the patient's general neurological status and life expectancy when selecting an appropriate treatment strategy.

Long-term outcomes after AVM treatment were generally good, especially for patients with complete AVM obliteration. Longer follow-up studies, such as those by Faye et al. [22] and Motegi et al. [15], provide evidence of the durability of both radiosurgery and microsurgical resection, with stable obliteration rates over time and a very low recurrence rate. However, the possibility of hemorrhage cannot be entirely ruled out over the patient's life expectancy. This was illustrated by a report of a hemorrhagic event 19 years post-treatment by Majewska et al. [17]. Resolution without intervention, though rare, has also been observed. For instance, Braileanu et al. [16] described a case of spontaneous obliteration in a small AVM, reminding physicians that observation can occasionally lead to a cure. These occurrences, however, are highly infrequent, and such a strategy should be adopted only in selected cases with favorable clinical profiles.

This review underscores the necessity of a multidisciplinary approach to managing thalamic AVMs, with neurosurgery, radiosurgery, and interventional radiology playing integral roles. Treatment should be individualized based on the patient's clinical presentation, AVM size and location, and the associated risks of intervention. While radiosurgery is often the preferred choice due to its minimally invasive nature, the associated risks of delayed complications and the need for long-term follow-up must be weighed against the more immediate but higher-risk outcomes of microsurgical resection.

Radiosurgery provides an overall good balance of efficacy and safety for most patients with thalamic AVMs, particularly those in whom surgical intervention is considered too risky. Microsurgical resection remains an important option for immediate resolution in selected cases, especially for large or hemorrhagic AVMs. Embolization, although less frequently utilized, represents an additional tool in specific cases. Further studies are needed to better define the long-term outcomes of conservative approaches and to explore, identify, and develop innovative and less invasive means for AVM treatment.

## Conclusions

Thalamic AVMs are among the most challenging entities in neurosurgical practice because of their deep location and proximity to important brain structures. The main emphasis of this systematic review is on the multidisciplinary approach, including microsurgery, stereotactic radiosurgery, and, in selected cases, embolization or conservative management. Radiosurgery presents a favorable balance between effectiveness and minimal invasiveness; at the same time, it carries risks of delayed complications, such as hemorrhage. While microsurgical resection has higher morbidity, it can be considered a preferred treatment for larger or hemorrhagic AVMs. Treatment strategies need to be meticulously designed based on individual patients' symptomatology and the characteristics of their AVM, including follow-up through the years and potential sequelae. Further studies are warranted to explore new treatment modalities and to analyze the outcomes of conservative management strategies for selected cases of thalamic AVMs.
